# Factors contributing to under-5 child mortality in Nigeria: A narrative review

**DOI:** 10.1097/MD.0000000000041142

**Published:** 2025-01-03

**Authors:** David B. Olawade, Ojima Z. Wada, Nicholas Aderinto, Aderonke Odetayo, Yusuff Adebayo Adebisi, Deborah Tolulope Esan, Jonathan Ling

**Affiliations:** a Department of Allied and Public Health, School of Health, Sport and Bioscience, University of East London, London, United Kingdom; b Department of Research and Innovation, Medway NHS Foundation Trust, Gillingham, United Kingdom; c Division of Sustainable Development, College of Science and Engineering, Hamad Bin Khalifa University, Qatar Foundation, Doha, Qatar; d Department of Medicine and Surgery, Ladoke Akintola University of Technology, Ogbomoso, Nigeria; e School of Nursing, Tung Wah College, Hong Kong SAR, China; f Nuffifield Department of Population Health, University of Oxford, Oxford, United Kingdom; g Faculty of Nursing Science, College of Health Sciences, Bowen University Iwo, Iwo, Nigeria; h Faculty of Health Sciences and Wellbeing, University of Sunderland, Sunderland, United Kingdom.

**Keywords:** health accessibility, health disparities, infant survival, mortality risk factors, pediatric mortality

## Abstract

Despite repeated efforts by the Nigerian government and the international community, under-5 child mortality remains alarmingly high in Nigeria. This narrative review aims to reassess the key factors contributing to this persistent public health challenge. A comprehensive search of peer-reviewed articles and reports published in English was conducted to identify and synthesize data on the factors predisposing Nigerian children under 5 to mortality. The review identifies multiple interrelated contributors, including socioeconomic, sociocultural, and demographic factors, inadequate access to healthcare services, an under-resourced healthcare system, and a shortage of qualified healthcare professionals. The high burden of communicable and preventable diseases also plays a significant role in under-5 mortality. To address these issues, targeted interventions such as improving healthcare access, strengthening the health system, and reducing poverty are essential. The findings underscore the urgent need for a coordinated, multi-sectoral approach to effectively reduce under-5 mortality in Nigeria and improve the health outcomes of vulnerable children. Government, healthcare providers, and communities must work together to address these concerns so that all children can access the care they need to live and flourish.

## 1. Introduction

**Figure 1.**
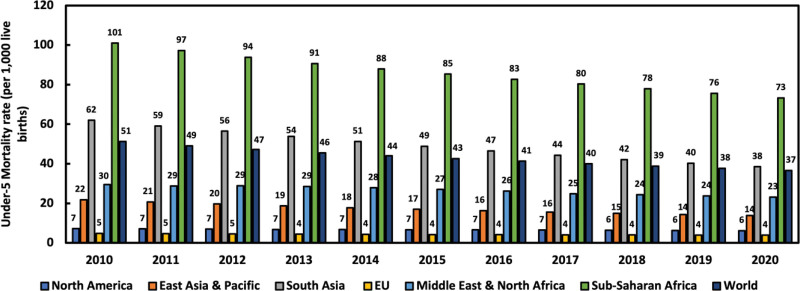
Under-5 mortality trends across different regions from 2010 to 2020.

Child mortality, especially under-5 mortality (U5M), is a significant global health issue and a key health indicator for countries all around the world. Between 1990 and 2020, there has been a significant decline in U5M from 12.6 million in 1990 to 5 million in 2020.^[1]^ This is equivalent to a 60% decline from 93 deaths per 1000 live births in 1990 to 37 deaths per 1000 live births in 2020.^[2]^ This decline has not been reflected equally in regions, and a child’s birthplace significantly impacts their survival chances.^[3–6]^ Compared to children born in high-income countries (5 deaths per 1000 live births), children born in low-income countries (67 deaths per 1000 live births) had over 10 times higher risk of dying before reaching the age of 5.^[3]^ Figure 1 shows the under-5 mortality rate across selected regions of the world for the past decade using World Bank data.^[7]^

The sub-Saharan Africa (SSA) region has the highest U5M globally.^[8–10]^ In 2021, over 80% of the estimated 5 million deaths of children under 5 years occurred in SSA and southern Asia, with approximately 78 deaths per 1000 live births in SSA.^[2]^ This rate is about 15 times higher than in Europe and North America and 19 times higher than in New Zealand and Australia.^[3]^ Most of these deaths are due to infectious diseases like measles, malaria, cholera, diarrhea, and respiratory infections. Even though these diseases are preventable and curable, the lack of efficient healthcare intervention strategies has resulted in continued and high U5M in SSA.^[7]^

**Figure 2.**
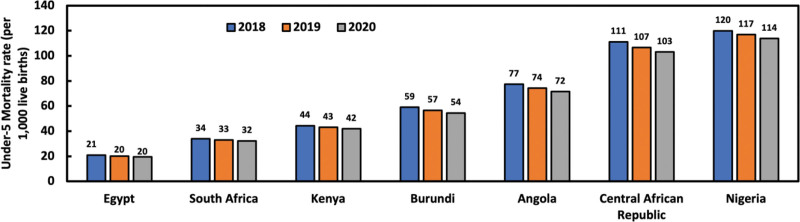
Under-5 mortality trends across selected African countries.

**Figure 3.**
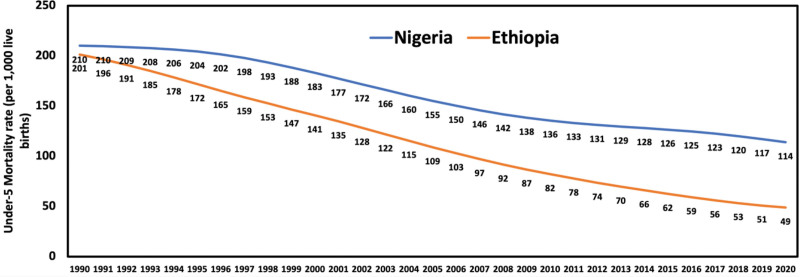
Under-5 mortality trends in Nigeria and Ethiopia from 1990 to 2020.

Childhood mortality is still a significant public health issue in Nigeria despite the marked decline in childhood deaths globally. Nigeria has the highest record of under-5 mortality in SSA.^[11]^ According to the World Health Organization (WHO), Nigeria, including 4 other countries (India, Pakistan, the Democratic Republic of Congo, and Ethiopia), accounted for half of all deaths among children under the age of 5 in 2020, with nearly a third of all deaths occurring in Nigeria (844,000 deaths) and India (783,000) alone.^[2]^ Although under-5 deaths have declined in recent years in Nigeria, progress is subpar, and the rate remains unacceptable. What sets Nigeria apart is its stark disparity in child mortality rates based on wealth. According to a UNICEF report, while many countries have seen the absolute gap between the richest and poorest households narrow since 1990, Nigeria continues to exhibit the largest disparities. In 2022, the under-5 mortality rate among the poorest households in Nigeria was 142 per 1000 live births, compared to 54 deaths per 1000 live births among the wealthiest households: a disparity of 88, the highest in the world.^[1]^ In 2021, under-5 mortality ranged from 52 deaths per 1000 live births in some regions to as high as 253 deaths per 1000 live births in others.^[1]^ This wealth and regional disparity contribute to Nigeria’s poor performance compared to other African nations. As shown in Figure 2, when compared to selected countries from North, East, and Central Africa, Nigeria lags significantly in reducing under-5 mortality.^[7]^ Figure 3 illustrates the time series from 1990 to 2020, highlighting that while Ethiopia and Nigeria had similar under-5 mortality rates of around 200 deaths per 1000 live births in 1990, Ethiopia has achieved significant progress, reducing its rate to 49 per 1000 live births in 2020. In contrast, Nigeria’s rate remains much higher at 114 deaths per 1000 live births.

**Figure 4.**
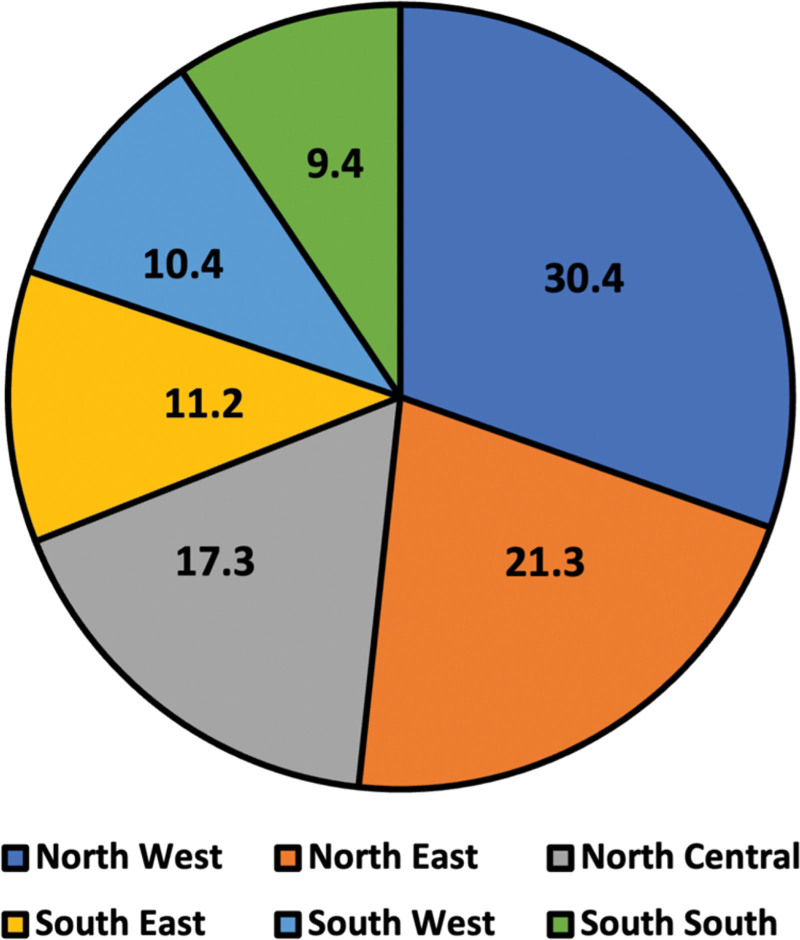
Under-5 mortality rates across the 6 geopolitical regions in Nigeria.

In addition, maternal age, like young mothers, often due to early childbearing age or older mothers, who may face increased health complications, including preterm birth or gestational hypertension, are at higher risk of losing their children before the age of 5.^[12,13]^ Additionally, multiple births, such as twins or triplets, have been linked to a high risk of prematurity, low birth weight, and congenital anomalies, which can contribute to a higher likelihood of under-5 mortality.^[14,15]^ Furthermore, low rates of exclusive breastfeeding, immunization, and lack of access to sanitary and clean water facilities have been identified as risk factors associated with under-5 mortality in Nigeria.^[13,16,17]^ These factors tend to be more prevalent in Northern Nigeria, thereby creating disparities in under-5 mortality rates between the Northern and Southern geopolitical zones. Figure 4 is a chart showing the distribution of under-5 mortality across Nigeria. The Sustainable Development Goal-3 aims to lower the under-5 mortality rate to 25 deaths per 1000 live births by 2030,^[18,19]^ which may not be met due to the slow progress towards reducing childhood mortality in Nigeria. Hence, we aim to provide an overview of the causes of Nigeria’s high under-5 mortality rate and propose possible solutions.

## 2. Materials and methods

This narrative review aimed to discuss the factors contributing to under-5 mortality in Nigeria. To achieve this, a search was conducted using different databases, including PubMed, Google Scholar, and relevant online sources. The search was limited to peer-reviewed articles published in English with no date restriction. The search terms used included “under-5 mortality in Nigeria,” “factors contributing to under-5 mortality in Nigeria,” “healthcare and under-5 mortality in Nigeria,” “maternal and child health in Nigeria,” and similar phrases. To be included in the review, articles had to meet the following criteria: be relevant to the topic of under-5 mortality in Nigeria; be published in English; be peer-reviewed; and provide original data or insights on the factors contributing to under-5 mortality in Nigeria. The selected articles were reviewed, and relevant data were extracted and organized according to the factors contributing to under-5 mortality in Nigeria. The extracted data were synthesized to identify the most reported factors contributing to under-5 mortality in Nigeria. The narrative review was organized according to these factors.

### 2.1. Limited access to healthcare

Under-5 mortality in Nigeria is influenced by poor access to healthcare. Access to primary care, hospitals, and specialized care is challenging for many children nationwide.^[20,21]^ Poorer health outcomes result from this lack of access, which means illnesses and injuries may be treated too slowly or inadequately. The problem is particularly severe in rural areas of Nigeria, where there are few or no healthcare facilities, and those that exist are frequently understaffed and underequipped.^[14,22,23]^ This causes children in rural locations to receive delayed or insufficient care, which raises their risk of morbidity and mortality.

The cost of services in Nigeria is a major impediment to children’s access to healthcare^[24]^ as many families struggle to pay for healthcare, especially private healthcare. This is especially troublesome for low-income families and people who live in rural areas, who frequently have limited access to government-funded healthcare facilities. Access to healthcare for children in Nigeria is also severely hampered by the lack of healthcare providers, especially in rural areas.^[25–28]^ This worsens the issue of limited access to healthcare by preventing children in these communities from having access to skilled medical providers.

The Nigerian government has developed several initiatives to improve children’s access to healthcare. These include the Primary Health Care Under One Roof initiative and the National Health Insurance Scheme,^[29–31]^ which seek to increase access to primary healthcare in rural and underserved areas. The National Health Insurance Scheme aims to provide health insurance coverage for low-income families.^[31]^ The government has also been attempting to enhance the overall healthcare system and increase spending on healthcare. To address the poor healthcare access that contributes to under-5 mortality in Nigeria, additional work must be done. Along with strengthening the healthcare system to ensure the availability of necessary drugs and equipment, increasing investment in healthcare infrastructure and human resources, especially in rural areas, and addressing socioeconomic barriers that restrict access to healthcare, such as poverty and illiteracy, are also needed.

### 2.2. Inadequate infrastructure

A significant factor in Nigeria’s high under-5 mortality rate is inadequate infrastructure. Lack of proper infrastructure, like sanitary and clean water, electricity, and roads, can make it challenging for healthcare providers to reach and deliver services to communities, particularly rural communities.^[26,32–35]^ This causes the care for children living in these communities to be delayed or insufficient, which raises their risk of illness and mortality. Nigeria is a serious impediment to access to healthcare.^[36,37]^ The lack of adequate roads and transportation infrastructure in many rural areas severely impedes healthcare access, making it challenging for families to reach medical facilities. Children who live in distant places and who frequently travel great distances to get healthcare services are most affected by this problem. Furthermore, the lack of sanitary and clean water facilities can bring about water-borne illnesses like diarrhea,^[33,38,39]^ which can be fatal to children if not promptly addressed. The risk of illness and mortality among children is further increased by poor sanitation,^[40]^ which can help infectious diseases spread.

The high under-5 mortality rate in Nigeria has prompted the government there to establish several policies and programs to address the issue. These include the National Rural Access and Mobility Project, which aims to increase rural access to healthcare by building and repairing rural roads and bridges,^[41]^ and the National Primary Health Care Development Agency, which strives to enhance primary healthcare services in the nation.^[42]^ The National Health Act, which aims to offer Universal Health Coverage and enhance the nation’s general healthcare system, was enacted by the government in 2014.^[31,43]^ Through initiatives like the Rural Water Supply and Sanitation Agency, which strives to offer safe drinking water to rural populations and enhance sanitation facilities, the government has also been trying to supply sanitary and clean water to improve hygiene in rural areas.^[44]^ On the other hand, there is still work to be done to address poor infrastructure as a contributing cause to Nigeria’s high under-5 death rate. This entails boosting spending on transportation and healthcare infrastructure, especially in rural areas, and addressing socioeconomic issues like education and poverty that obstruct access to healthcare. Furthermore, it is critical to concentrate on offering long-term, sustainable solutions rather than just treating the symptoms of the problems to enhance overall health results.

### 2.3. Dearth of healthcare providers

The long-standing challenge of shortage of healthcare providers is another influencing factor of the high under-5 mortality rate, which is particularly acute in rural regions of Nigeria.^[25,45,46]^ This deficit continues to be a significant obstacle to reducing under-5 mortality. A system that is overburdened and pressured due to a lack of healthcare providers also influences the standard of care. According to the WHO, the recommended ratios for doctors and nurses are 1:1000 and 1:500, respectively.^[47,48]^ However, Nigeria has a deficit, with a doctor-patient ratio of 1:5000 and a nurse-patient ratio of 1:3000.^[47]^ This deficit affects children in rural regions, who are unable to access trained healthcare providers, resulting in delayed or suboptimal care and poor health outcomes.^[48]^

Over the years, the Nigerian government has launched policies and programs to address the shortage of healthcare providers, which includes expanding the number of healthcare providers, enhancing their education and retention, raising the healthcare budget, and enhancing the healthcare system.^[49,50]^ However, progress in addressing this shortage has been modest. Government funding for healthcare education and training programs, incentives for healthcare providers to work in remote and underserved areas, enhanced working conditions, and pay are needed.^[51]^ To guarantee that the healthcare workforce is sustainable and able to satisfy the people’s demands, the government must also address underlying socio-economic issues, such as poverty and a lack of education, that contribute to the scarcity of healthcare providers. Governmental intervention should also focus on improving the nation’s primary healthcare system, which serves as the first point of contact for most people seeking medical attention.^[52]^ By making primary healthcare investments, additional healthcare providers could be sent to underserved regions and rural areas, increasing access to healthcare services and enhancing children’s health outcomes.

Involving the private sector in the delivery of healthcare services, particularly in rural communities, is another potential strategy. This could involve public-private collaborations, which can aid in boosting the availability of health workers and enhancing the standard of care. A multifaceted strategy that targets the root causes of the shortage and concentrates on offering long-term, sustainable solutions is needed to address the trend of a scarcity of healthcare providers as a contributing factor to Nigeria’s high under-5 death rate.

### 2.4. Socio-economic factors

The health and wellbeing of Nigerian children are directly impacted by socioeconomic conditions.^[53]^ Tackling these issues is, therefore, essential to lowering the country’s under-5 mortality rate. Poverty may make it more challenging for a family to pay for essentials like food, sanitary and clean water, and medical treatment.^[32,53]^ Low-income families are more likely to have malnourished children, which can result in stunted growth and increased disease vulnerability. Children’s development and health may suffer long-term consequences because of this. The availability of sanitary and clean water facilities is a crucial socioeconomic element.^[33]^ Additionally, restricted access to education might restrict parents’ access to information and resources to help them make informed decisions about their children’s health.^[54]^ Furthermore, education can lift families from poverty and enhance their socioeconomic standing. The government must adopt a multifaceted strategy that addresses the root causes of poverty and limited access to education to address these socioeconomic concerns as a role in Nigeria’s high under-5 death rate. This entails funding social safety initiatives like cash transfers and food assistance, expanding access to education, and giving families instruction and support to help them improve their standard of living. Access to adequate housing and sanitary and clean water facilities is crucial for enhancing child health outcomes. Overall, reducing under-5 mortality in Nigeria requires tackling socio-economic variables. Improving the health and wellbeing of children paves the way for a better future for the next generation.

### 2.5. Socio-cultural factors

Socio-cultural factors significantly influence under-5 mortality in Nigeria. Traditional beliefs, customs, and habits that may impact children’s health and well-being can be among these influences.^[55]^ Traditional attitudes and behaviors surrounding childbirth and childrearing have the potential to raise the mortality rate among children under 5 in Nigeria.^[45,55]^ Many mothers choose to give birth at home with traditional birth attendants rather than in a hospital setting with trained birth attendants.^[56]^ Poor maternal and neonatal outcomes, including a higher risk of mother and newborn death, may result from this.

The use of traditional healers and reliance on herbal medicine is a common sociocultural factor that influences increased mortality in children under 5 in Nigeria. Many mothers in rural community settings sought the services of traditional healers rather than modern medicine, particularly for childhood illnesses such as diarrhea, malaria, and respiratory infections.^[57]^ This traditional practice and behavior delay the early diagnosis of these illnesses, which are primary contributors to mortality in those under-5s.

The traditional practice of female genital mutilation is another sociocultural element that raises the death rate for children under 5 in Nigeria. For both girls and women, this practice has detrimental health effects. These include an increased chance of maternal death after childbirth.^[58]^ Cultural traditions around nutrition may also harm children’s health. For instance, some traditional practices and beliefs could restrict the use of contemporary baby feeding methods, such as breastfeeding, which can result in malnutrition and poor health consequences for infants.^[55,59]^

Polygamy and large family size are other sociocultural factors contributing to under-5 mortality in Nigeria. In polygamous families with many children, resources are usually stretched thin, leading to insufficient nutrition, inadequate access to healthcare, and a lack of proper care for younger children.^[60]^ This increases the likelihood of preventable diseases and malnutrition, both of which contribute to high mortality rates.

The government must adopt a multifaceted strategy that targets the underlying reasons for these habits if it is to address these socio-cultural elements. Teaching local populations about the dangers of traditional beliefs and practises and collaborating with residents and traditional leaders to advance positive cultural norms and behaviors can help reduce child mortality. Involving and teaching the community about the value of healthcare and appropriate childcare practices can also aid in reducing long-standing habits that are harmful to children’s health. To ensure that the cultural context is considered and that the community accepts the programmes, communities should help develop the design and implementation of health initiatives. Involving and teaching the community about the value of healthcare and ethical childcare methods can also aid in reducing the cultural and traditional behaviors that harm children’s health.

### 2.6. Insufficient funding

Over the years, there has been a persistent pattern linking inadequate funding to Nigeria’s high under-5 mortality rate. The Nigerian government has regularly committed a low percentage of its budget to healthcare, which has left a gap in the infrastructure and resources needed to deliver basic healthcare services to children. Nigeria has one of the lowest healthcare expenditures as a percentage of GDP globally, at about 3%, according to data from the WHO.^[7]^ This is substantially below the minimum 5% of GDP for developing nations. Additionally, much of the healthcare budget is frequently allocated to tertiary care and urban areas, underfunding primary healthcare and rural areas.^[52,61]^ This pattern of underfunding has resulted in children’s lack of access to healthcare services. In addition, a lack of funding for healthcare facilities and staff has resulted in a shortage of medical employees, supplies, and equipment, which may compromise the standard of care.

The Nigerian government has undertaken several policies and programmes over the years to address inadequate funding to reduce the under-5 mortality rate in the nation.^[62]^ However, there has been little success in addressing the pattern of inadequate finance, and it continues to be a major obstacle to reducing under-5 mortality in Nigeria. The government must invest more in healthcare and devote a larger portion of the national budget to reverse this trend, especially in primary healthcare. Furthermore, it is critical to concentrate on offering long-term, sustainable solutions rather than just treating the symptoms of underfunding.

### 2.7. High disease burden

High under-5 mortality in Nigeria is principally caused by a high disease burden. Malaria, malnutrition, and pneumonia, which are important sources of morbidity and mortality in children, are among the many treatable and avoidable diseases that are prevalent in the nation.^[63–65]^ The high prevalence of malaria in Nigeria, which is a primary cause of death for children under the age of 5, is one of the country’s biggest health issues.^[66,67]^ Malaria is most prevalent in rural areas. Many children lack access to necessary preventive measures, such as bed nets, as well as quick and efficient medical care when they become ill.

Malnutrition, a prominent cause of death for children under 5, is a key factor in the under-5 mortality rate in Nigeria.^[68]^ Malnourished children are more prone to infections and illnesses and have a higher risk of passing away from conditions that can be prevented and treated. Nigeria has a high disease burden, with pneumonia ranking second among causes of mortality in children under the age of 5.^[69]^ Bacteria, viruses, or other microbes can cause pneumonia, a lung infection. In Nigeria, a large number of children lack access to necessary preventive measures, such as immunisations, as well as quick and efficient medical care when they become ill. According to WHO/UNICEF immunization coverage data, Nigeria had around 15% (2.1 million) of children who had not received routine immunization (also known as zero-dose children) in 2023, making it the highest population of zero-dose children globally.^[70]^ For example, around 57% (3 million) of children did not receive the first dose of the measles vaccine in 2022,^[71]^ while the coverage for the second dose of measles was only 38% in 2023.^[72]^ Other routine immunizations with low coverage in 2023 were rotavirus last dose and yellow fever, with 49% and 59% coverage, respectively.^[72]^ This shows that immunization coverages in Nigeria still fall below the recommended percentage for herd immunity, and this can contribute to a high disease burden leading to mortality in children under 5 years of age.

## 3. Limitations of the review

This narrative review has several limitations that should be noted. First, the review relied exclusively on English-language articles and reports. This language restriction may have limited the inclusion of locally conducted studies or reports published in other languages, which might have provided unique regional insights, especially from areas where English is not the primary language. Additionally, the review’s findings are influenced by the quality and availability of data from existing literature. Many included studies may have inconsistent reporting standards, incomplete data, or differing metrics. These variations could affect the synthesis and accuracy of identified mortality factors, leading to possible gaps in understanding the complete picture of under-5 mortality causes in Nigeria.

Furthermore, as a narrative review, this study does not utilize systematic or meta-analytic approaches that might have provided quantitative synthesis or minimized potential biases. The findings are, therefore, not as robust as those from more structured reviews and may be subject to the biases of individual studies included. The review also focuses on broad socioeconomic, sociocultural, and health system factors without delving deeply into how these interact within specific Nigerian regions or communities. This broad focus may result in missed insights on regional disparities, where factors contributing to child mortality may vary significantly.

Another limitation is the temporal relevance of the data sources used. Some data may reflect past trends and not fully capture recent changes or government interventions in Nigeria’s public health landscape. As a result, some findings may lack current relevance due to the evolving nature of health policies and interventions in Nigeria. Lastly, as a narrative review, this study does not contribute original data, limiting its ability to offer real-time insights or establish a baseline for longitudinal studies. Future studies could address these limitations by employing a systematic review approach, including a broader linguistic criterion, and ideally incorporating primary data collection to provide a more comprehensive and up-to-date understanding of the factors influencing under-5 mortality in Nigeria.

## 4. Conclusion

Over the past ten years, Nigeria’s under-5 mortality rate has been slowly declining, although it is still very high. Increasing access to healthcare, enhancing the standard of care, and fortifying the healthcare workforce, particularly in rural and underserved areas, are just a few of the initiatives the nation needs to keep putting into practice and scaling up. Tackling the root causes of the high rate of under-5 mortality in Nigeria will necessitate a multifaceted strategy. It also entails strengthening the healthcare workforce, enhancing the standard of care delivered in healthcare facilities, removing socioeconomic and sociocultural barriers, and increasing healthcare funding. This includes enhancing access to healthcare and maternal and child health services, particularly in rural areas. Achieving this goal will also require coordination and cooperation between the government, NGOs, and the private sector.

## Author contributions

**Conceptualization:** David B. Olawade.

**Writing – original draft:** David B. Olawade, Ojima Z. Wada, Nicholas Aderinto, Aderonke Odetayo, Yusuff Adebayo Adebisi, Deborah Tolulope Esan, Jonathan Ling.

**Writing – review & editing:** David B. Olawade, Ojima Z. Wada, Nicholas Aderinto, Aderonke Odetayo, Yusuff Adebayo Adebisi, Deborah Tolulope Esan, Jonathan Ling.
